# Effective clinical supervision of allied health professionals: a mixed methods study

**DOI:** 10.1186/s12913-019-4873-8

**Published:** 2019-12-31

**Authors:** David A. Snowdon, Michelle Sargent, Cylie M. Williams, Stephen Maloney, Kirsten Caspers, Nicholas F. Taylor

**Affiliations:** 1 Peninsula Clinical School, Peninsula Health, Monash University, Frankston, VIC 3199 Australia; 2 Department of Speech Pathology, Peninsula Health, Frankston, VIC 3199 Australia; 3 Department of Physiotherapy, Monash University, Frankston, VIC 3199 Australia; 4 Department of Allied Health, Peninsula Health, Frankston, VIC 3199 Australia; 5 Allied Health Clinical Research Office, Eastern Health, La Trobe University, Level 2/5 Arnold Street, Box Hill, VIC 3128 Australia

**Keywords:** Supervision, Professional education, Physiotherapists, Occupational therapists, Podiatry, Speech pathology, Dietetics, Psychology, Social Workers

## Abstract

**Background:**

Clinical supervision is recommended for allied health professionals for the purpose of supporting them in their professional role, continued professional development and ensuring patient safety and high quality care. The aim of this mixed methods study was to explore allied health professionals’ perceptions about the aspects of clinical supervision that can facilitate effective clinical supervision.

**Methods:**

Individual semi-structured interviews were conducted on a purposive sample of 38 allied health professionals working in a metropolitan public hospital. Qualitative analysis was completed using an interpretive description approach. To enable triangulation of qualitative data, a quantitative descriptive survey of clinical supervision effectiveness was also conducted using the Manchester Clinical Supervision Scale (MCSS-26).

**Results:**

Three main themes emerged from qualitative analysis: Allied health professionals reported that clinical supervision was most effective when their professional development was the focus of clinical supervision; the supervisor possessed the skills and attributes required to facilitate a constructive supervisory relationship; and the organisation provided an environment that facilitated this relationship together with their own professional development. Three subthemes also emerged within each of the main themes: the importance of the supervisory relationship; prioritisation of clinical supervision relative to other professional duties; and flexibility of supervision models, processes and approaches to clinical supervision. The mean MCSS-26 score was 79.2 (95%CI 73.7 to 84.3) with scores ranging from 44 to 100. MCSS-26 results converged with the qualitative findings with participants reporting an overall positive experience with clinical supervision.

**Conclusions:**

The factors identified by allied health professionals that influenced the effectiveness of their clinical supervision were mostly consistent among the professions. However, allied health professionals reported using models of clinical supervision that best suited their profession’s role and learning style. This highlighted the need for flexible approaches to allied health clinical supervision that should be reflected in clinical supervision policies and guidelines. Many of the identified factors that influence the effectiveness of clinical supervision of allied health professionals can be influenced by health organisations.

## Background

Clinical supervision is recommended for allied health professionals for the purpose of supporting them in their professional role, continued professional development and ensuring patient safety and high quality care [1–4]. Clinical supervision involves an experienced allied health professional guiding the practice and development of a less experienced allied health professional [2–4]. Clinical supervision also aims to bridge the gap in professional experience between the supervisee and supervisor, ensuring patient care and supervisee well-being is not affected by inexperience [2–4].

The term ‘allied health’ is used to describe health professionals other than nursing and medical professionals [5, 6]. Allied health professionals can be further classified into three categories: therapy (e.g. physiotherapists, occupational therapists, social workers); diagnostic and technical (e.g. optometrists, audiologists, orthotists); and scientific (e.g. pharmacists, medical scientists) [6]. In Australia, allied health professionals are tertiary educated and registered with a professional board or association [5]. As such, they are qualified to act as primary care therapists where a referral is not required from a medical professional to receive allied health services [5]. Therefore, they have the capacity to work across the public and private health settings in private clinics, outpatient health clinics and hospitals [5].

The allied health professions have adopted Proctor’s model of clinical supervision to guide them in their clinical supervision practice [7]. Proctor’s model of clinical supervision describes how health professionals can be supported in the formative, restorative and normative domains of practice [8]. The formative domain refers to the development of skills that are specific to the health professional’s role; the restorative domain refers to supporting the professional through the emotional burden of their professional role; and the normative domain refers to health professional compliance with standards of care and organisational policy and procedures [8]. Therefore, effective clinical supervision should support allied health professionals in all three of Proctor’s domains [9].

Evaluations of the effectiveness of clinical supervision to support allied health professionals in their professional role have focused on the therapy professions working in metropolitan and regional Australian public health care settings (e.g. hospitals, health care centers) [10–12]. These evaluations have found that clinical supervision is broadly effective for allied health professionals and that the level of effectiveness varies between the individual professions [10–12]. Based on health professional report using the Manchester Clinical Supervision Scale (MCSS-26) [13] Dawson and colleagues found that, on average, clinical supervision was effective in a small group of 30 allied health professionals [10]. In a larger cohort of allied health professionals (*n* = 196) Snowdon and colleagues found that, on average, clinical supervision was effective for the social work, psychology and occupational therapy professions [11]. While in the physiotherapy, podiatry, dietetics and speech pathology professions, the effectiveness of clinical supervision was uncertain [11]. These findings have also been replicated in another study with physiotherapists reporting significantly less effective clinical supervision than occupational therapists [12]. These findings highlight the importance of exploring factors that contribute to effective clinical supervision across the allied health professions.

There has been qualitative and quantitative exploration of the different factors influencing the effectiveness of clinical supervision of allied health professionals predominantly working in regional Australian public health care settings. Allied health professionals who choose their supervisor, work in community settings or have spent less than 1 year in their role typically report higher levels of effectiveness [14, 15]. Longer and more frequent supervision sessions have also been shown to positively influence the effectiveness of clinical supervision [15]. Interviews with allied health professionals identified the importance of organisational policies and procedures, and a positive clinical supervision culture as facilitators of effective clinical supervision [16, 17]. Clear guidelines on the conduct of clinical supervision (e.g. frequency, duration and roles of supervision) ensure that supervision occurs frequently rather than ad hoc [17], while a strong culture of clinical supervision facilitates access to technology that enables supervision to be delivered to therapists who practice remotely [16]. Finding time for clinical supervision and difficulty establishing an appropriate supervisee-supervisor match have been reported as barriers to effective clinical supervision [16–18]. Competing clinical duties and the geographical barriers that exist in remote health care settings contribute to the issue of finding time [16, 17]. While finding an appropriate supervisor to match the supervisee’s experience levels/interests was also challenging due to limited breadth and depth of some professions (e.g. small department size) and difficulties in finding a supervisor who understands the contextual factors of practising in a remote setting (e.g. sole practitioner, broad range of caseload and considerable time spent travelling) [17, 18]. However, it is unclear whether these factors are similar for allied health professionals practicing in larger health services in metropolitan settings.

The aim of this mixed methods study was to explore allied health professionals’ perceptions about the aspects of clinical supervision that can facilitate effective clinical supervision. Exploring this question enables insight on the aspects of clinical supervision that are effective and will guide how to facilitate effective clinical supervision for all allied health professionals.

## Methods

### Study design

A mixed methods study design was used. Qualitative research methods using semi-structured interviews explored allied health professionals’ experiences with clinical supervision and aspects of supervision perceived as effective. An interpretive description methodological approach was used to obtain a better understanding of the phenomenon of clinical supervision and to generate knowledge that could be applied in the future supervision of allied health professionals [19, 20]. To enable triangulation of qualitative data, a quantitative descriptive survey of clinical supervision effectiveness was also conducted using the Manchester Clinical Supervision Scale (MCSS-26) [13]. Peninsula Health Ethics Committee approved this research (LNR/45695/PH-2018) and all participants provided written informed consent.

### Participants

Eligible participants were allied health therapy professionals from the physiotherapy, occupational therapy, social work, dietetics, psychology, podiatry and speech pathology professions, working in hospital-based services across four hospital sites for a public health network in Melbourne, Australia. Allied health professionals working solely in community-based services were ineligible to participate due to the variation in their supervision structure in this health care organisation.

Eligible allied health professionals were selected for participation using purposive sampling to ensure the sample was representative of the department’s diversity. This included allied health professionals from a variety of professions, clinical specialities, hospital sites and levels of experience. To enable sufficient representation of the department’s diversity and to likely reach data saturation with no new themes emerging, we planned to interview 38 allied health professionals on their experience receiving clinical supervision.

### Clinical supervision policy and procedure

Allied health professionals participate in clinical supervision practice as guided by the health network guideline. This guideline recommends that clinical supervision fulfils the three functions of Proctor’s model; that allied health professionals receive support in the development of professional skills, meeting organizational requirements and managing the emotional burden of practice [8]. It places emphasis on the practice of reflective supervision, where the allied health professional is required to reflect on their work experience and deconstruct both the cognitive and emotional aspects of their work [21, 22]. It also encourages other models of clinical supervision such as the direct supervision model where the supervisor observes clinical practice and assists with patient management [23]. Allied health professionals within this health organisation are also required to receive supervision from a more senior professional of the same profession. The frequency of supervision sessions is dictated by level of experience; junior or intermediate professionals are required to receive supervision fortnightly and senior professionals monthly.

### Data collection

Semi-structured interviews were conducted by one researcher (DS). The interviewer had no clinical or supervisory relationship with any of the participants. An interview guide (Table 1) was used to ensure that relevant topics were addressed. The interview guide had been previously used in a study investigating the aspects of clinical supervision that are effective for physiotherapists [24]. It was further piloted on four allied health professionals, one each from the speech pathology, occupational therapy, podiatry and social work professions. The purpose of piloting the interview guide was to ensure the questions remained appropriate across the allied health professions. No changes were made to the original interview guide following this process.

**Table 1:** Semi-structured Interview Guide

Topic	Sample Questions
What is the role of clinical supervision?	What do you feel is the purpose of supervision? What areas of your professional role do you feel supervision should support you in?
What is your experience of effective clinical supervision?	What activities do you do during supervision that you feel supports you in your professional role? What do you feel are the enablers to these activities? What do you feel are the barriers to these activities? What other things have you experienced during supervision in the past that you found effective/didn’t find effective?
What are your ideals regarding clinical supervision?	Are there activities that you’re not doing during supervision at the moment that you would like opportunity to participate in? Why do you feel that you currently don’t have the ability to participate in these opportunities? Is there anything else that you feel could happen to better support you with clinical supervision?

Preceding each interview participants completed the MCSS-26 [13]. Participants rated the level to which they agreed with each item on a 5-point Likert scale, ranging from ‘strongly disagree’ to ‘strongly agree’. The MCSS-26 consists of six sub-scales that can be summed to provide a domain summary score for each of Proctor’s domains. The sum of all six sub-scales provides a total score ranging from 0 to 104 and a score of ≥73 indicating effective supervision [13]. The scale has undergone Rasch analysis and has demonstrated evidence of validity in the allied health professions [25].

### Data analysis

Interviews were audiotaped and transcribed verbatim. Participants reviewed the transcripts to ensure they were an accurate representation of their perceptions [26]. Where participants felt that the transcript did not accurately represent their perceptions, participants amended the transcript. All participants confirmed that transcripts were an accurate representation of their perceptions. Four participants returned transcripts with minor corrections/clarifications relating to spelling errors and inaccurate transcription of individual words. Following corrections each transcript was assigned a number for further analysis.

The interpretive description approach was used in this study to focus on the reality of clinical supervision practice with the objective of producing findings which could positively impact on its practice and effectiveness [19, 20, 27]. Interpretive description provides a flexible structure for describing a phenomenon (effective clinical supervision) and understanding it from the perspective of those experiencing it (allied health professionals) [19, 20, 27]. Interpretive description consists of two philosophical underpinnings: 1) reality is subjective, constructed, and contextual; and 2) the researcher and participant interact to create research understandings [20]. Inductive thematic analysis was used as an analytic approach as it is consistent with the interpretive description methodology [19]. This ensured that themes were generated from the researchers’ interpretation of participants’ experiences with clinical supervision.

Rigor of data analysis was enriched through use of a reflective diary to document researcher observations and experiences during the interview [28]. Three researchers (DS, MS, NT) coded transcripts independently using NVivo qualitative data management software [29]. Consensus between all three researchers on the emerging themes was achieved collaboratively through discussion. The researchers (DS and MS) then re-read transcripts to selectively search for data related to the identified themes (selective coding). Themes were confirmed by researchers (DS and MS) checking transcripts following discussion, during which no new themes arose, suggesting saturation was achieved [30]. Links and relationships between the confirmed themes were established and an overarching theory was formulated. Trustworthiness was established by triangulation with MCSS-26 scores (methodologic triangulation) and between researchers (investigator triangulation) [31].

## Results

### Participants

Thirty-eight allied health professionals participated in this research; seven (18%) physiotherapists, nine (24%) occupational therapists, seven (18%) social workers, four (11%) dietitians, two (5%) psychologists, four (11%) podiatrists and five (13%) speech pathologists. Eleven participants (29%) were grade 1 (junior), seventeen (45%) were grade 2 (intermediate) and ten (26%) were grade 3 (senior) allied health professionals. The majority of participants were female (*n* = 34, 90%) with a mean (SD) age of 31 (6) years. Twenty participants (53%) had supervisor responsibilities within the department. On average participants had received clinical supervision for 6 years (range 1 to 18 years) and typically participated in monthly clinical supervision sessions of 30 to 60 min duration. Supervisors had been allocated to all participants.

### Themes

**Fig. 1:**
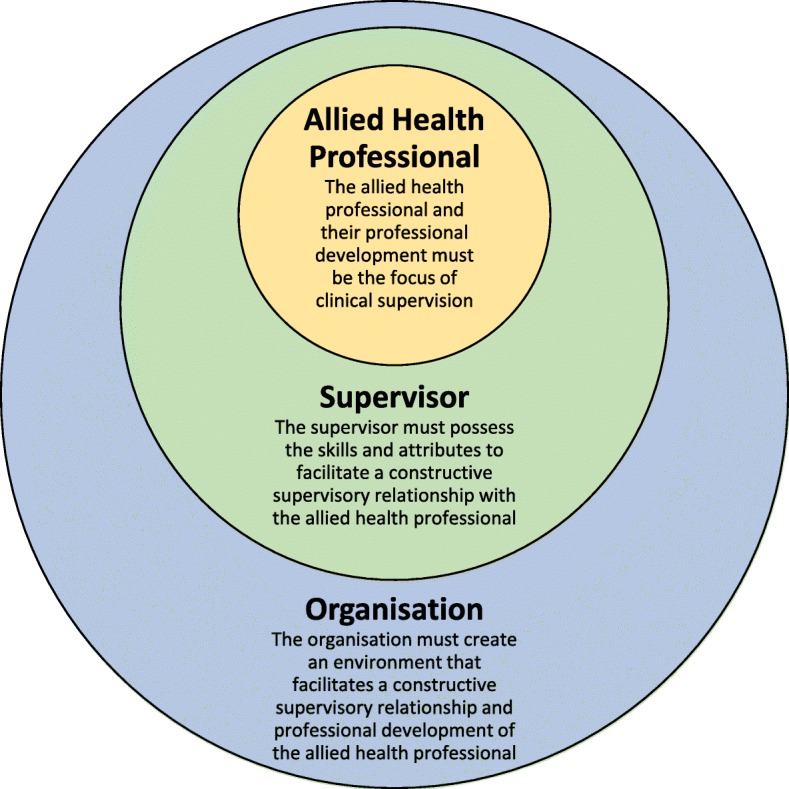
Effective clinical supervision of allied health professionals

Three main themes emerged from the qualitative analysis (Fig. 1). Allied health professionals reported that clinical supervision was most effective when their:

1. professional development was the focus of clinical supervision
2. supervisor possessed the skills and attributes required to facilitate a constructive supervisory relationship
3. organisation provided an environment that facilitated this relationship and their professional development.

Figure 1 shows how these three main themes interact. At the centre of the model is the focus on professional development. The supervisor skills/attributes and organisational environment should facilitate this focus.

On the outside is the organisational environment, which highlights the importance of the organisation supporting both the professional development of allied health professionals and ensuring supervisors are skilled, and prepared to support this development.

Three subthemes also emerged within each of the main themes: the importance of the *supervisory relationship*, *prioritisation* of clinical supervision relative to other professional duties and *flexibility* of supervision models, processes and approaches to clinical supervision.

### Allied health professional development should be the focus of clinical supervision

Allied health professionals reported that clinical supervision was most effective when it supported and facilitated their professional development. When clinical supervision addressed their professional development allied health professionals were more likely to report *prioritising* clinical supervision. Clinical supervision was less effective and a lower *priority* when there was more focus placed by the supervisor on the operational component of their role such as reporting on their own performance without addressing their learning needs.

‘*The purpose of supervision is to reflect on your own skill level and also interpersonal skills and look at developing both as a person and clinician within the workplace.*’ P30, occupational therapist.

To ensure their professional development was facilitated through clinical supervision the allied health professionals reported that clinical supervision should be *flexible* and adaptable to their learning style. As such, allied health professionals used clinical supervision in a variety of ways to support their professional development. There were some similarities between and within the professions. For example, in addition to reflecting on their professional performance with their supervisor, professions with a ‘hands on’ clinical role (i.e. physiotherapists, occupational therapists, podiatrists and speech pathologists) reported using a direct model of clinical supervision where they treated patients alongside their supervisor or observed their practice.

‘*Any time there was something strange or unusual or something we didn’t see every day my supervisor would call me in and we’d actually treat it together. That hands-on experience as well as through discussion of cases was really valuable*.’ P13, podiatrist.

‘*It was really useful to watch my supervisor manage a patient. Not just hear about what she would do, but see how she does it*.’ P14, speech pathologist.

These professions reported a focus on their competence in performing a particular skill or task within their professional role.

‘*I might bring my handover and systematically go through each patient and explain what I’m doing with them to my supervisor to get that feedback that I’m on the right track.*’ P33, occupational therapist.

In contrast, the social work and psychology professions reported using clinical supervision as a time to reflect on their professional and personal development, which required them to explore how their own values and beliefs may impact on their interactions with colleagues/patients and patient management.

‘*It’s the steps I took and how can I improve upon that but it’s also how did my personal and my professional values affect this? What did that look like for me?*’ P21, social worker.

All professionals reported the importance of receiving clinical supervision and guidance from their supervisor external to scheduled sessions. This form of clinical supervision was usually referred to as ‘informal’ and enabled the professional to address arising issues and receive timely feedback. This also facilitated trust in the *supervisory relationship*. This form of clinical supervision was not a substitute for the formal scheduled sessions, and the scheduled time allowed the allied health professional time to focus on the ‘bigger picture’ of their professional development.

‘*It’s quite a dynamic quick environment … you can’t necessarily wait till the next week to sit down and discuss your tricky patient because you need to pretty much have a plan instantly about what you’re going to do, which is I guess the benefit of the informal supervision.*’ P38, physiotherapist.

The majority of allied health professionals reported that supporting their professional development also had positive benefits for their wellbeing. As their skills improved they could be more confident that they were providing the best possible care for their patients and performing at a high standard within their professional role. This reassurance reduced their stress levels and improved their job satisfaction. However, the counselling professions emphasised the need to debrief on patient interactions or presentations that were particularly complex and stressful. They reported that there is often emotional burden associated with the management of patients with complex needs and this requires debriefing to ensure no detrimental impact on their mental health or wellbeing. Therefore, in addition to supporting professional development, the counselling professions also required an additional level of emotional support.

‘*All areas where I was seeing risk and also loss, quite a bit of bereavement so just having someone who could work through that with you and hear that, another social worker just saying “Yeah, I get it.*” P21, social worker.

### The supervisor should possess the skills and attributes required to facilitate a constructive supervisory relationship

Allied health professionals reported that the supervisor’s skills and attributes were crucial to facilitating their professional development and a constructive *supervisory relationship*. First and foremost it was identified that supervisors should respect the allied health professional, value the supervision process and invest time into facilitating the allied health professional’s development.

‘*I think definitely the supervisor is important and you want them to be able to feel that the process is important as well and also to spend time developing you.*’ P4, dietitian.

Professionals also preferred the supervisor was experienced and skilled in their professional role. Professionals reported that they were more likely to *prioritise* clinical supervision and seek their supervisor’s professional opinion and guidance when their supervisor had expertise in their field of practice.

‘*You’ve also got to be able to look up to the person that’s supervising you in terms of their skill level. I think it’s got to be the skill level that you’re either striving to achieve or that is on par with yours so that they can provide useful supervision.*’ P36, occupational therapist.

Effective communication was identified as another skill that was desirable in supervisors. Specifically, allied health professionals valued supervisors who clearly outlined the expectations within the supervisory relationship and who could provide constructive feedback.

‘*I’ve had a few different supervisors over the years and the times where I’ve been able to establish a better rapport with the supervisor is the early sessions being about getting to know each other; getting to know each other’s learning styles and preferred methods of communication and setting some of the ground rules and expectations early on.*’ P5, social worker.

Allied health professionals also preferred supervisors who were *flexible* in their supervision style. These supervisors tailored their approach to the professional’s learning style rather than selecting an approach that was convenient or focused only on meeting the organisational requirements for supervision.

‘*My supervisors have been great in accommodating that extra time despite what the guideline says about how frequently we should be meeting. It’s been on a necessity-basis*.’ P3, social worker.

Allied health professionals reported that supervisors who were disinvested, inexperienced in their professional role, poor communicators or inflexible were detrimental to the *supervisory relationship* and that clinical supervision was less effective and harder to *prioritise* under such circumstances.

### The organisation should provide an environment that facilitated a constructive supervisory relationship and development of allied health professionals

Allied health professionals reported that the organisation plays a key role in ensuring that the environment facilitates their professional development and the practice of clinical supervision. Allied health professionals explained the importance of working within a department or organisation where clinical supervision was valued and its purpose clearly outlined. Allied health professionals reported that working in departments that valued clinical supervision also enabled *prioritisation* of clinical supervision relative to other professional duties (e.g. clinical care). This ensured time for clinical supervision was protected and sessions were regularly scheduled into professionals’ diaries.

‘*It’s made very clear by our manager and it’s modelled by the staff here, that clinical supervision is a priority, and it will take priority over clinical work if it has to.*’ P24, speech pathologist.

‘*First and foremost I’d probably say support from your managers to allow you to first have the time and also to instil that belief that it’s important to have supervision*.’ P8, physiotherapist.

There were also several practical steps identified that organisations could take to ensure the environment was suitable. These steps included ensuring a confidential space is available, providing resources such as documentation templates and a supervision contract between the supervisor and allied health professional, and ensuring co-location of the supervisor and supervisee. It was also important that the organisation provided *flexibility* in how these resources are used to ensure they fit the allied health professional’s learning style.

‘*You do need somewhere really private because at times you’re handling confidential, sensitive issues.*’ P20, physiotherapist.

‘*I like to follow a guideline during supervision sessions so we’ve got a template that we go through and I find that helps keep me on track*’ P33, occupational therapist.

‘*I think, starting off, signing a supervision contract is quite a valuable tool to use.*’ P24, speech pathologist.

‘*Constructive feedback is actually quite relevant if the person works with you day to day.*’ P35, dietitian.

Allied health professionals who were supervised by their direct line manager identified the dual role of manager/supervisor as a barrier to effective clinical supervision. They reported that there was often conflict within this *supervisory relationship* and they were less likely to identify their weaknesses or areas for improvement. The allied health professionals acknowledged the limitations of supervisor allocation within a hierarchical organisational structure, but identified alternative arrangements the health organisation could implement to facilitate a more constructive supervisory relationship. These included peer supervision or being allocated a supervisor external to the health service.

‘*My supervisor is also my line manager, so I’d feel a little bit defensive or protective maybe bringing up some cases where I feel that maybe I haven’t done my best work.*’ P37, podiatrist.

Allied health professionals also identified that the organisation could provide formal training for both supervisees and supervisors to ensure a uniform competency of supervision skills across departments. They believed that this would help to facilitate constructive *supervisory relationships*, especially professionals who were from the non-counselling professions (i.e. physiotherapy, dieticians, occupational therapists, podiatrists, speech pathologists) and who regularly rotated workplace and supervisors.

‘*I think that everyone engaging in supervision should have some form of training in supervision. I think that would help everyone be more committed and on-board to the value of supervision.*’ P25, speech pathologist.

‘*We should have some competencies around providing supervision and then probably having some kind of education that people are expected to attend.*’ P30, occupational therapist.

### Effectiveness of clinical supervision

The mean MCSS-26 score was 79.2 (95%CI 73.7 to 84.3) with scores ranging from 44 to 100. Twenty-five participants (66%) scored an MCSS-26 score of ≥73 indicative of effective clinical supervision. Participants who rated above this score were five (71%) physiotherapists, five (56%) occupational therapists, six (86%) social workers, two (50%) dietitians, two (100%) psychologists, three (75%) podiatrists and two (40%) speech pathologists. These findings converged with the qualitative findings with participants reporting an overall positive experience with clinical supervision and were solution focussed in addressing the barriers to effective clinical supervision. Participants rated clinical supervision least effective in the normative domain of the MCSS-26 (Table 2). Within the normative domain participants scored lowest in the finding time subscale. This finding also converged with the qualitative findings with participants identifying possible solutions to the issue of finding time. Specifically they identified having skilled supervisors and organisations/managers/supervisors that valued supervision as key factors that assisted in prioritising clinical supervision over other professional duties.

**Table 2:** Manchester Clinical Supervision Scale (MCSS-26) Scores

	Score Range	Median (Range) (Raw Score)	Mean (95%CI) (Raw Score)	Mean (95%CI) (out of 100)
Total MCSS-26	0–104	81 (44 to 100)	79.2 (73.7 to 84.3)	76.1 (71.0 to 81.2)
Formative Domain	0–28	22 (11 to 28)	22.5 (20.3 to 23.7)	80.3 (74.2 to 86.3)
Improved Care/Skills	0–16	13 (6 to 16)	12.4 (11.2 to 13.6)	77.5 (70.2 to 84.7)
Reflection	0–12	10 (5 to 12)	10.1 (9.4 to 10.8)	84.0 (78.4 to 89.6)
Restorative Domain	0–40	32 (14 to 39)	31.2 (28.8 to 33.7)	78.1 (71.9 to 84.3)
Trust/Rapport	0–20	16 (6 to 20)	15.6 (14.2 to 16.9)	77.8 (71.2 to 84.3)
Supervisor Advice/Support	0–20	16 (8 to 20)	15.7 (14.1 to 17.3)	78.4 (70.6 to 86.3)
Normative	0–36	25 (18 to 36)	25.5 (23.7 to 27.2)	70.7 (65.8 to 75.6)
Importance/Value of CS	0–20	17 (13 to 20)	17.1 (16.2 to 18.0)	85.3 (80.7 to 89.8)
Finding Time	0–16	8 (1 to 16)	8.4 (7.0 to 9.8)	52.5 (43.9 to 61.1)

Physiotherapists, occupational therapists, dietitians and speech pathologists reported a lower median and score range for the reflection subscale (median: 9, range: 5–12) compared to social workers and psychologists (median: 11, range: 7–12). These results converged with qualitative findings that social work and psychology professions primarily viewed clinical supervision as a reflective process, while the other professions reported a need for direct supervision in combination with reflective practice.

All professions scored highly in the improved care/skills subscale. This result converged with the qualitative finding that participants primarily viewed clinical supervision as a professional development activity with a focus on developing skills. However, it diverged with the finding that participants did not identify improved patient care or outcomes as a primary aim of clinical supervision.

## Discussion

Allied health professionals identified three factors required for effective clinical supervision: the focus of clinical supervision should be on the professional development of the allied health professional, the supervisor should possess the skills and attributes required to facilitate a constructive supervisory relationship and the organisation should provide an environment that facilitates this relationship and development of the allied health professional. The supervisory relationship, prioritisation of clinical supervision and flexibility in supervision approach were also identified as key areas that must be addressed for effective clinical supervision. The factors identified by allied health professionals that influenced the effectiveness of their clinical supervision were mostly consistent among the professions, and should be considered by allied health professionals and health organisations to guide clinical supervision policy and practice.

The organisational and supervisor factors identified by allied health professionals in our study are similar to findings in rural settings [16]. These factors may be influenced to facilitate effective clinical supervision through initiatives such as the development of supervision guidelines, separating clinical supervision from line management, and supervisor training for allied health professionals [17, 32, 33]. However, unlike previous evaluations our study identified a focus on professional development as a key factor for facilitating effective clinical supervision. This is an important consideration for health organisations who have standardised the practice of clinical supervision across the allied health professions for the purpose of clinical governance and ensuring safe, high quality care [1]. While supervision should perform a role in ensuring high quality care, initiatives that are too focused on clinical governance may ignore the role of clinical supervision to facilitate professional development.

Similarities between the professions in the factors that influence the effectiveness of clinical supervision support the development of one clinical supervision policy for allied health professionals rather than individual policies for each profession [34]. In Australia, clinical supervision policies and guidelines exist for many of the professions [34, 35]. This is thought to contribute to the variability in the practice of clinical supervision and may explain some of the variability of effectiveness of clinical supervision in the allied health professions [35]. It has been argued that the development of a universal clinical supervision policy for the allied health professions would improve the overall quality of clinical supervision but may be difficult given the absence of an agreed definition of clinical supervision and consensus on how it should be practised [34]. Our findings show that despite this diversity, the factors that influenced the effectiveness of clinical supervision were consistent among the professions. Therefore, a universal policy/guideline that encourages flexibility in regards to the model of clinical supervision that is used may be beneficial for allied health professionals.

While many of the factors that influenced the effectiveness of clinical supervision were consistent across the professions there was some variability in how the professions used clinical supervision to support their professional development. This variability is reflected in our findings within the flexibility subtheme and may be explained by the difference in allied health professional roles and profession specific preferred learning styles [36]. Occupational therapists, speech pathologists and physiotherapists have been found to prefer a kinaesthetic learning style indicating a preference for learning through practice or situations [37]. In contrast, social workers and psychologists have been shown to favour more reflective learning styles [38, 39]. This may explain the use of a direct supervision model in the professions with a ‘hands on’ clinical role and sole use of the reflective model in the counselling professions. Similarly, the preference of social workers and psychologists to debrief and analyse their feelings related to practice may be explained by a preference in these professions to learn through reflecting on their feelings and emotions experienced during their practice [38, 39]. Other professions, such as physiotherapy and occupational therapy, appear to require learning activities that enable them to analyse and synthesise information and form objective decisions [40, 41].

The differences in the practice of clinical supervision between the professions should be acknowledged and supported by organisational clinical supervision guidelines [34]. In the allied health professions there has been a focus on reflective supervision; notably Proctor’s model of clinical supervision emphasises the importance of reflective practice [42, 43]. Organisations that encourage and support the practice of a direct model in addition to a reflective model of clinical supervision may better meet the learning needs of all allied health professions. This is demonstrated by the physiotherapists in this study who reported the opportunity to participate in direct supervision and overall effectiveness of clinical supervision with this model. Furthermore, the majority (73%) of physiotherapists in this study reported effective clinical supervision, albeit in a relatively small sample. This result contrasts previous evaluations where less than 50% of physiotherapists reported effective supervision [11, 35]. Therefore, it is likely important that supervision guidelines acknowledge the differences between the professions in how they use supervision to facilitate their professional development and promote a flexible approach to supervision [34].

Ensuring that guidelines acknowledge direct models of supervision, involving observation of supervisees’ clinical practice, might have a positive effect on patient safety and care [44, 45]. Some allied health professionals do not believe that observation of supervisees’ clinical practice is an aspect of clinical supervision [46]. This is despite the role of clinical supervision as a form of clinical governance [1], and evidence showing an association between direct models of supervision and improved patient safety and care [44, 45]. Clear organisational guidelines that outline the role of clinical supervision in ensuring high quality patient care may positively influence the acceptability and practice of the direct models of clinical supervision by all allied health professionals, and enhance patient care [46].

The MCSS-26 results showed that finding time was a barrier to effective clinical supervision and is consistent with previous findings [7, 11, 12, 14–17]. Allied health professionals who report difficulty finding time for clinical supervision also report higher levels of emotional exhaustion [47]. Hence, this issue is a concern for allied health departments and may have wider implications including a negative impact on patient care [10]. Allied health professionals in our study demonstrated a solution focused approach to the barrier of finding time identifying that they were more likely to make time for clinical supervision when managers and supervisors prioritised clinical supervision. Therefore, solutions to this problem may involve managers allocating protected time for supervision, and ensuring that supervisors are skilled and accountable in their role as supervisor [48, 49].

Although clinical supervision has been shown to be effective for ensuring the safety and quality of patient care the allied health professionals in this study did not report this as an aim of effective clinical supervision [44, 45]. Instead, allied health professionals reported their motivation for clinical supervision was primarily their own professional development. Improved professional development might be expected to result in better patient outcomes. However, recent research has shown allied health professionals’ perceived effectiveness of clinical supervision in a rehabilitation setting was not associated with improved patient outcomes [50]. This indicates the support perceived as effective may not necessarily influence patient care [50]. This is important for health organisations to consider when implementing clinical supervision for the purpose of improving the quality of patient care. Ideally, clinical supervision should be effective for the health professional and their patient care, but to achieve this, consideration needs to be given to both the professional development of the health professional and oversight of their clinical practice to ensure it is adherent to clinical practice guidelines.

This mixed methods study includes the first qualitative evaluation of the effectiveness of clinical supervision of allied health professionals working in a metropolitan hospital setting. The study also benefits from the perspectives of a sample of allied health professionals from a variety of professions who reported varying levels of effectiveness of clinical supervision. In addition, this study is the first to develop a model that can be used by allied health professionals and health organisations to guide clinical supervision policy and practice. There are limitations to this study that may affect the generalisability of the findings. Participants consisted of allied health professionals working in an inpatient hospital setting and results may not be generalisable to allied health professionals working in community settings or to allied health professionals working in the science or diagnostic areas. The sample of 38 allied health professionals could be considered small given the number of professions (*n* = 7) they represented. This may have limited the ability of this study to identify profession-specific factors in the aspects of clinical supervision that are effective at supporting them in their professional role for some professions with small numbers, such as psychologists (*n* = 2). However, across the group we found that we reached saturation with the common themes that emerged. Also, this study only considers the effectiveness of clinical supervision to support allied health professionals in their professional role and does not provide evidence on the effectiveness of clinical supervision to ensure quality of care and patient safety [50].

## Conclusion

The factors identified by allied health therapy professionals that influenced the effectiveness of their clinical supervision were mostly consistent among the professions. Allied health professionals perceived their clinical supervision effective when it focused on their professional development. They identified the supervisor’s skills and the support provided by the organisation played a significant role in facilitating effective clinical supervision. Allied health professionals reported using models of clinical supervision that best suited their profession’s role and learning style. This highlighted the need for flexible approaches to allied health clinical supervision and clinical supervision policies and guidelines should reflect these. Many of the identified factors that influence the effectiveness of clinical supervision of allied health professionals can be influenced by health organisations.

## Data Availability

The dataset generated and analysed during this study is not publicly available due to conditions of ethical approval. However, a de-identified version of the data is available from the authors upon reasonable request.
